# Growth of Plasmonic Nanoparticles for Aging Cask-Matured Whisky

**DOI:** 10.1021/acsanm.2c03406

**Published:** 2022-10-06

**Authors:** Jennifer Gracie, Francesco Zamberlan, Iain B. Andrews, Brian O. Smith, William J. Peveler

**Affiliations:** † School of Chemistry, University of Glasgow, Glasgow G12 8QQ, U.K.; ‡ The Scotch Whisky Research Institute, Edinburgh EH14 4AP, U.K.; § School of Molecular Biosciences, University of Glasgow, Glasgow G12 8QQ, U.K.

**Keywords:** whisky, maturation, aging, sensing, reduction, gold nanoparticles

## Abstract

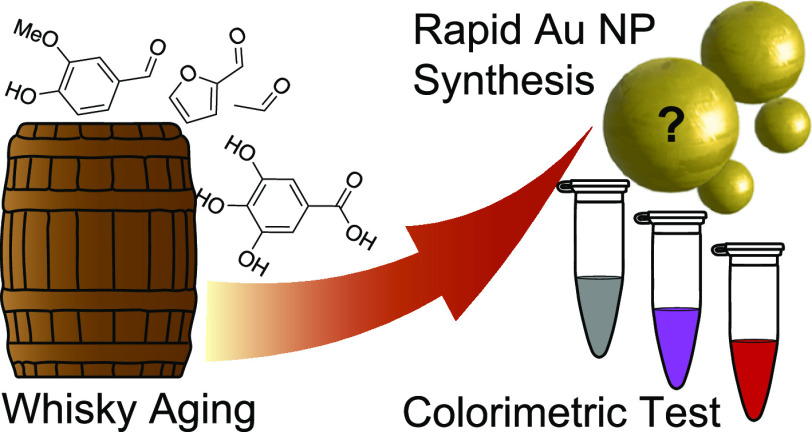

The maturation of spirit in wooden casks is key to the production of whisky, a hugely popular and valuable product, with the transfer and reaction of molecules from the wooden cask with the alcoholic spirit imparting color and flavor. However, time in the cask adds significant cost to the final product, requiring expensive barrels and decades of careful storage. Thus, many producers are concerned with what “age” means in terms of the chemistry and flavor profiles of whisky. We demonstrate here a colorimetric test for spirit “agedness” based on the formation of gold nanoparticles (NPs) by whisky. Gold salts were reduced by barrel-aged spirit and produce colored gold NPs with distinct optical properties. Information from an extinction profile, such as peak position, growth rate, or profile shape, was analyzed, and our assay output was correlated with measurements of the whisky sample makeup, assays for key functional groups, and spiking experiments to explore the mechanism in more detail. We conclude that age is not just a number, that the chemical fingerprint of key flavor compounds is a useful marker for determining whisky “age”, and that our simple reduction assay could assist in defining the aged character of a whisky and become a useful future tool on the warehouse floor.

## Introduction

Whisky is a valuable global spirit, predominantly aged in oak casks to give a unique flavor profile. As an example, Scotch whisky, matured for at least 3 years in oak casks, is the world’s number one internationally traded spirit, with exports of £4.5 billion in 2021.^1^ The flavor of whisky arises from congeners—chemicals left in the spirit after distillation, absorbed from the cask wood, and transformed from interactions between spirit, wood, and oxygen over extended periods of time (years to decades). Many of the cask extracted congeners arise from the alcoholysis of lignin in the wooden cask during the aging process.^2^ Numerous factors impact the aging of spirit in a cask and the congeners released including, the initial spirit, or “new make”, composition: the source of wood and openness of grain; production of the cask (size, shape, and treatment, e.g., charring); the number of times the cask has been used before; the fill level and access to oxygen; and how long the spirit resides in it.

Once aged, the spirit from various casks is blended and bottled to create the finished product. The most prized whiskies are aged for decades in casks, increasing their flavor profile and expense dramatically. Many less-expensive, younger whiskies are often blended with a small portion of aged spirit to add depth of flavor, and researchers are exploring ways to chemically recreate this “aged” flavor profile.^3,4^ Thus, simply knowing the age of a single cask is not enough to judge how the contents have chemically aged, and how it might fit into a final product. Constant sampling of casks by highly experienced master blenders is required to monitor the progress of whisky aging, across hundreds or thousands of casks. This laborious work, coupled with the risk of fraud, where unaged/immature spirits are artificially colored and passed off as older products,^5^ means there is a desire for a rapid test of whisky agedness. Such a test would detect the key chemical changes to the new make spirit that arise from contact with the oak casks, and enable a blender to understand quickly how an individual cask is behaving or how “aged” a whisky contributing to a blend might appear.

To avoid relying solely on the nose and palette of the master blender, instrumental approaches have been taken to quantify the congeners present in aged whisky samples. Gas chromatography (GC) is used to identify volatile congeners such as higher alcohols, while high-performance liquid chromatography (HPLC) is used to examine non-volatile congeners.^2,6^ By measuring the increase of cask-related congeners and changes in the spirit composition, knowledge on the aging process can be gleaned, and HPLC or GC alongside mass spectrometry (HPLC-MS or GC-MS) has become the gold standard for analyzing whisky. The quantification of many congeners has also been demonstrated using ^1^H NMR spectroscopy, with maturation-related congeners having limits of detection between 1 and 5 μM.^7,8^ However, such tools are rarely available on the warehouse floor, or within budget for smaller distilleries, so lower cost approaches are desirable to gain a holistic picture of whisky aging.

Both mid infra-red and Raman spectroscopy, combined with multivariate analysis, have been applied to this end^9,10^ and can be used through optically transparent glass bottles.^11^ Simpler still are colorimetric or fluorometric sensing arrays based on molecular chromophores,^12,13^ metallic nanoparticles (NPs),^14^ and fluorescent polymers,^15^ and these have all been applied to distinguish and differentiate between (“fingerprint”) different whiskies and other spirits (e.g., Chinese liquor “baijiu”).^16,17^ Such methods can be portable and low cost, and with a well “trained” database can identify specific bottles/batches of spirit. However, in many cases, little information is extracted on the underlying sample chemistry that the array is “fingerprinting”. Notably, Anslyn and Wiskur built a sensor array with a more targeted molecular approach, creating receptors for known aromatic acid congeners such as gallic acid and ellagic acid and used UV–visible spectroscopy (UV–vis) to approximate spirit agedness through the quantitation of these compounds.^18^

Plasmonic metallic NPs have been employed as sensors in many complex mixtures thanks to the sensitivity of their strong plasmonic absorption to the local environment. For example, sensor arrays based on the growth or shape change of Au NPs have been applied in biomedical^19,20^ and food and drink analysis. Forest et al. recently demonstrated a gold NP aggregation assay to classify taste profiles and detect undesirable off-flavors in maple syrup in a simple portable test.^21^ NPs have also been suggested for use in a colorimetric assay for detecting reducing sugars in liquids. Brasiunas et al. demonstrated the detection of such compounds in carbonated soft drinks, milk and saliva through the mixing of preformed gold NP seeds, reductants, and stabilizing ligands in liquid samples and analyzing the plasmon produced.^22^

**Scheme 1:**
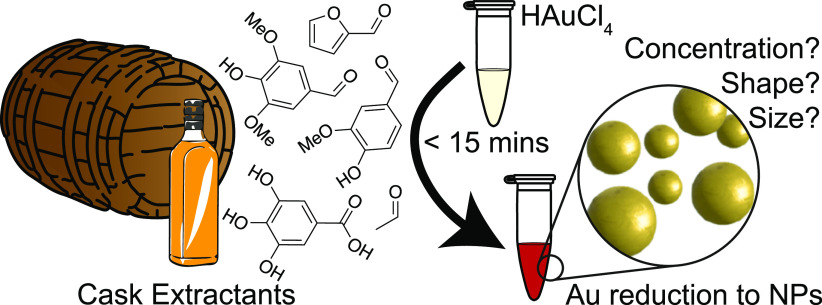
Overview of this Work, Using Wood Cask Extractants that Indicate the Agedness of Whisky in Cask to Synthesize Au NPs, with Particle Properties Being Indicative of the Reduction Process

Inspired by the needs and solutions described above, and by previous work showing the role of reductive compounds such as ketones and catechols in the formation of Au NPs,^19,23^ we demonstrate here a colorimetric sensor based on templated plasmonic NP growth for measuring the agedness of whisky (Scheme 1). The formation of gold NPs is induced by the whisky itself without the need for additional reagents, and the reaction occurs at room temperature over minutes. By comparing the plasmonic response across various aged samples, we show that this assay can indicate the reductive potential of a whisky and in turn give an indication of its agedness and production style. By analyzing the chemical makeup of the samples, we link the shape, size and rate of Au NP formation to the spirit’s reducing power (presence of aldehydes, phenols, and catechols) and complex chelating chemical content (e.g., tannic acid), via correlative analysis and spiking experiments. This simple, on-site, and inexpensive test could aid distillers in deciding whether maturing whiskies are ready to bottle or blend, prior to time-consuming tasting sessions. More broadly, improved understanding of the chemistry underlying Au NP reduction assays will improve their application in a wide range of analytical challenges.

## Results and Discussion

### Whisky Reduction Produces Au NPs

When whisky is mixed with an aqueous Au^3+^ solution, Au NP growth is observed. The reaction occurs at room temperature, with results observable by eye within minutes: the solution changes from a yellow/amber color to pink/red once the Au NPs form. The Au NP growth can be quantified over time using extinction spectroscopy.

The gold concentration and ratio of whisky to gold for the reaction was initially optimized (Figure S1), and a 100 μL total volume with a 1:1 ratio of neat whisky to gold solution was found to work well (final gold concentration of 0.125 mM in each sample). Higher concentrations of HAuCl_4_ were found to change the particle extinction at redder wavelengths, and in extreme cases inhibit Au NP formation altogether. Larger additions of whisky (>50% of the sample) tended to only influence the final extinction intensity rather than the peak position.

**Figure 1:**
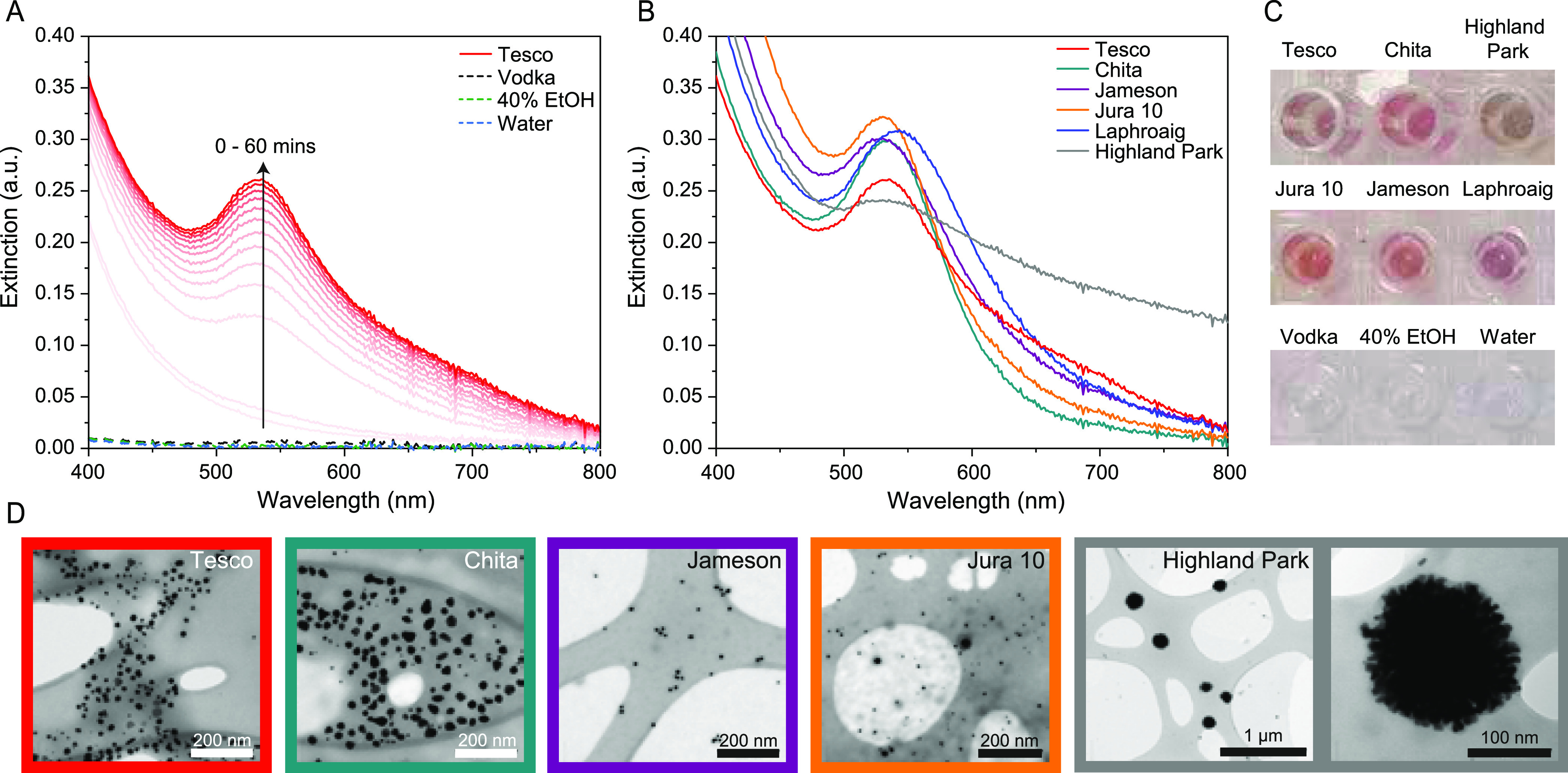
(A) Extinction spectra of Au NP formation over 1 h using Tesco whisky and control samples. Triplicate samples scanned in 5 min intervals, background water reference subtracted, and replicates averaged prior to plotting. (B) Extinction spectra of Au NP formation at 1 h time point using various whisky brands. Triplicate samples scanned at 1 h time point, background water reference subtracted, and replicates averaged prior to plotting. Additional samples are shown in the Supporting Information. (C) Image of sample wells with Au NPs formed from mixing 50 μL of 0.25 mM Au^3+^ solution and 50 μL of neat whisky (various brands) or controls (vodka, 40% EtOH, water). (D) TEM images of resulting Au NPs from select samples at 1 h growth. Border colors correspond to panel B.

For the model system, supermarket-own brand whisky was purchased from Tesco (Special Reserve Whisky, a blend of single malt and grain whiskies, aged for at least 3 years). The extinction spectra show the formation of Au NPs beginning after 10 min and continuing over a 1 h measurement period (Figure 1A). The emergence of a plasmon band at 535 nm indicated NP growth, with a λ_max_ that is characteristic of spherical Au NPs. The transmission electron microscopy (TEM) image (Figure 1D, red box) confirms the largely spherical Au NP formation. The control samples (Au^3+^ solution added to water, EtOH solution, and neat vodka, all shown as dashed lines) showed no evidence of NP formation over 1 h or indeed much longer periods of time. These negative controls highlight that the congeners from the whisky aging process are essential for reducing the gold salt, and not the alcohol content alone. Even in distilled spirits such as vodka, which contain some residual congeners from the fermentation process but are not cask aged, no Au NPs form.

To demonstrate generality, a range of other whiskies from around the globe were studied for their reducing potential and particle formation, and all the samples analyzed gave Au NPs on testing, despite varying age, country of origin, cask type, and barley/corn/grain source materials (Figures 1B and S2 and Table S1). Most samples produced spherical or spheroidal Au NPs, evidenced by plasmon resonance λ_max_ around 530–535 nm and the red color (Figure 1C), with a relatively narrow full width at half-maximum. TEM of selected samples confirmed the similar morphologies of the Au NPs, with some samples giving more or less faceted or spheroidal morphologies, and size varying (Figures 1D and S7 and Table S3). Samples with redder λ_max_ values (Tesco, Chita) had more anisotropic particles and a larger particle size (Figure S8). Another sample (Jura 10) was noted for having a small median Au NP size, but with a few much larger particles. Several samples gave much broader, almost featureless absorption, indicative of larger, more complex shapes or aggregates. In particular, Highland Park whisky produced Au NPs with a very broad plasmon band and corresponding distinct grey color (Figure 1C). On examination by TEM, the morphology was found to be large supraparticles (∼80 nm, Figure 1D, gray boxes) with a distinctive rough surface. This particle shape may have arisen from smaller Au NPs aggregating in a controlled manner or formed during the reduction and growth process itself, resulting in the irregular, “pom–pom”-like surface (Table S3). The kinetic data (Figures S3 and S5) show the growth of the Highland Park-reduced Au NPs over a 1 h period. Between 5 and 10 min appears to be the window in which Au NP formation is initially detectable, and by 10 min, the broad plasmon profile is already established and only the extinction intensity increases thereafter. Based on the marked differences of the plasmon peak position and width between samples, we hypothesize that the concentration and nature of the congeners present in each whisky sample influence the particles produced, both through rate of nucleation and growth, as well as surface capping. The differences in rate of growth could be further quantified by logistic fits of plasmon growth (Figures S5 and S6), with different samples producing very different profiles.

The reduction of Ag^+^ to plasmonic Ag NPs using whisky was also investigated, as a lower cost alternative to gold. The results closely mirrored those of the Au study (Figure S4), with the metal salt solution reduced by whisky samples to form Ag NPs (λ_max_ ≈ 416 nm) and the control samples showing no NP formation. However, to achieve similar by-eye observable results, the metal salt concentration had to be much higher (>1 mM), and the reduction reaction was slower, requiring hours and days instead of minutes, offsetting any cost benefits. Attempts to create copper NPs by reduction of Cu^2+^ with whisky were not successful.

### Whisky Cask Aging Influences Au NP Formation

**Figure 2:**
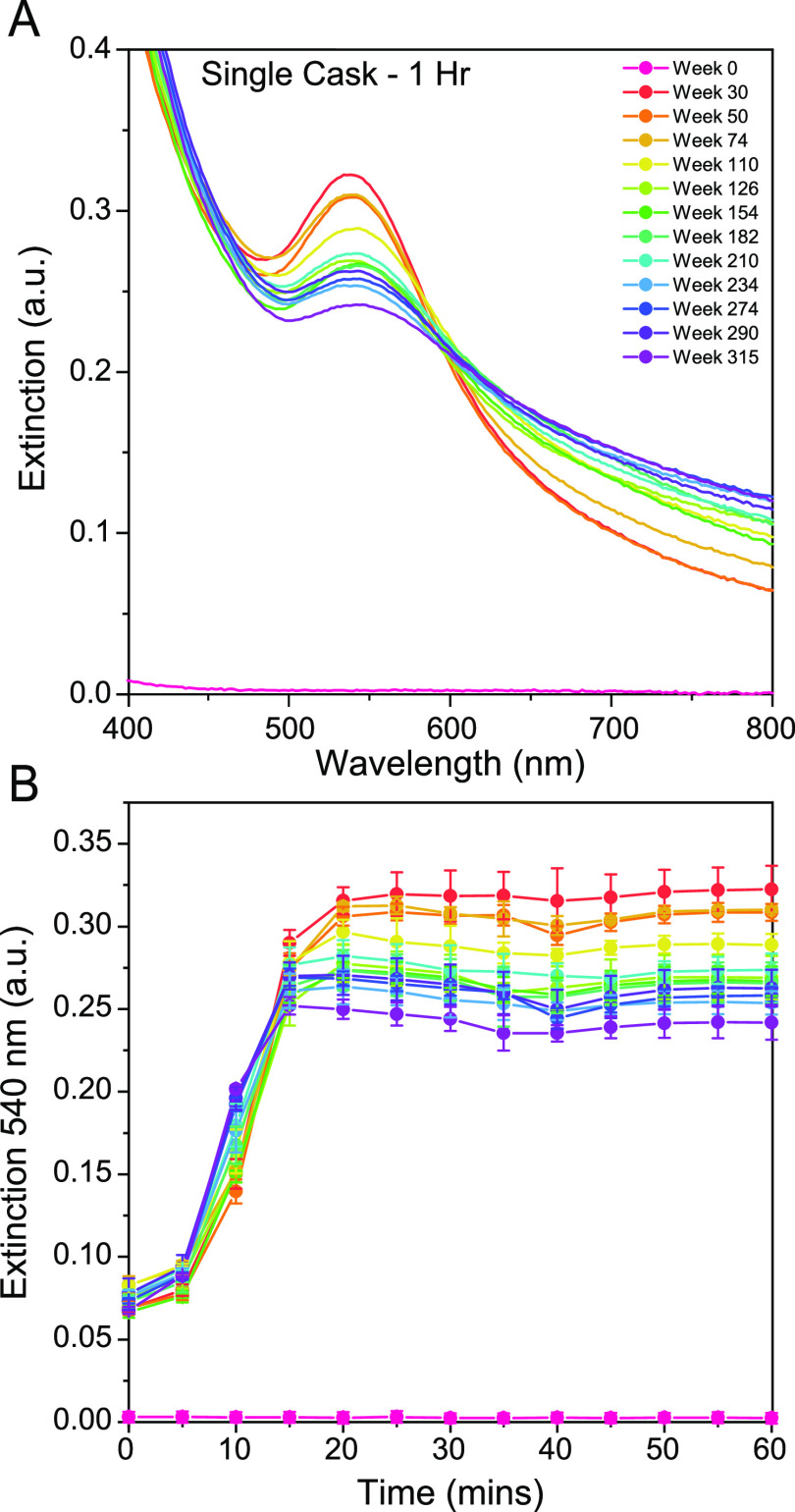
(A) Extinction spectra of 6 year single cask study. Spectra recorded at 1 h of reduction for each aged sample, background water reference spectrum subtracted, and three replicates averaged prior to plotting. (B) Growth of the 540 nm plasmon band vs time over 1 h for each aged sample.

To further our hypothesis that the formation of Au NPs might indicate the agedness of a spirit, we repeated our study on a longitudinal sample set from a single cask (malted barley spirit aged in an oak first-fill American Standard Barrel). Samples were kindly supplied by the Scotch Whisky Research Institute and were extracted at circa 6-monthly time points over a 6 year period, alongside a sample of the un-aged “new-make” spirit. The reducing potential of each sample was then tested as above and presented in Figure 2, with each whisky sample corrected to 40% alcohol by volume (ABV) prior to the addition of the Au^3+^ solution, to account for changes to spirit strength over time from ethanol evaporation (the loss of the “angel’s share”) and to allow a direct comparison to the commercial samples, which are all c. 40% ABV. In order to test neat samples directly, for example, at cask strength, an adaptation of the sample and reagent volumes and concentration in the assay may be necessary to isolate the effects of increased congener concentration and changes to congener composition between samples of different ages (Figure S1).

The spectra recorded are shown in Figure 2A. The new make spirit behaved as the vodka above, producing no discernible Au NPs, but the first whisky time point sample (30 weeks in cask) produced distinctive Au NPs with a λ_max_ at ≈540 nm. The most mature sample extracted at 315 weeks produced Au NPs with a similar λ_max_ at ≈540 nm but with a much broader plasmon band into the infrared, with samples moving from the former profile to the latter as they aged in cask. This plasmon band shape was distinct from many of the other whisky samples (Figures 1 and S2) but not completely dissimilar. This trend in the extinction spectra of the 6 year sample series suggests more and different congeners from the oak cask leach into the whisky during longer periods of maturation, altering the observed rate of reduction (Figures 2B and S5 and Table S2) and surface stabilization of the Au NPs. We hypothesize that the use of a first fill cask here will have accelerated that process of aging the spirit, thanks to the greater concentrations of lignin-derived compounds in the wood that have not been previously extracted.

### Whisky Congeners Influence Reducing Power

**Figure 3:**
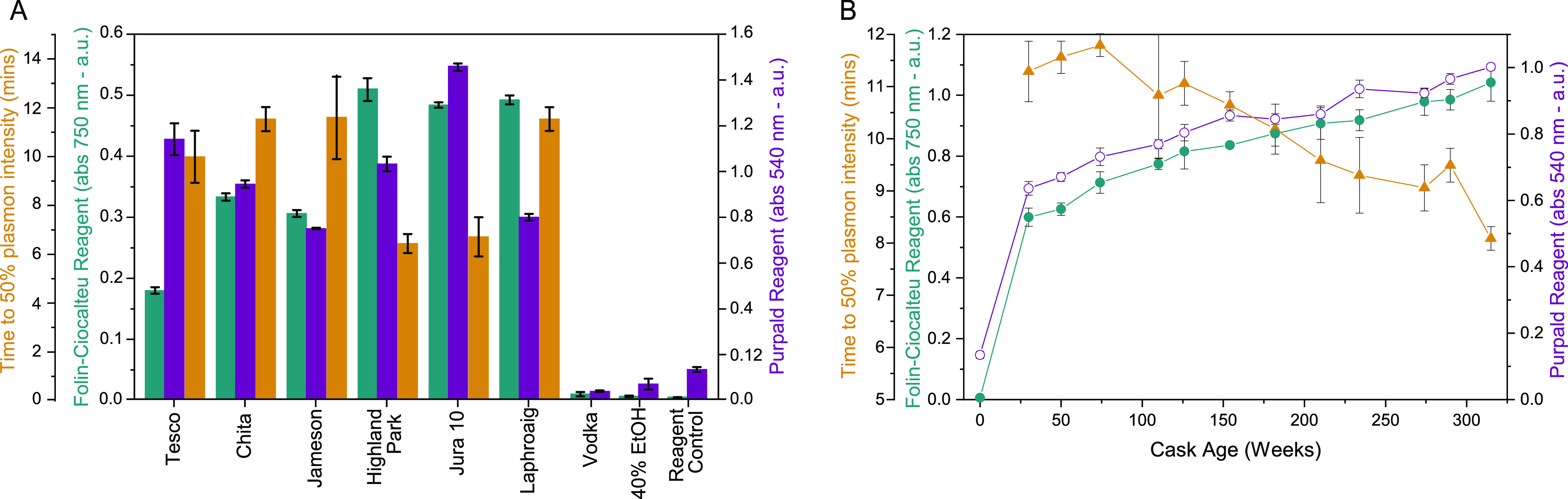
(A) Absorbance values (au) at λ = 750 nm for the FC assay (left in teal), time to 50% plasmon intensity as per a logistic fit of the data (*x*_0_ left in orange) and absorbance at λ = 540 nm for the purpald assay (right in purple) for different whisky brands and control samples. (B) Absorbance values (au) at λ = 750 nm for the FC assay (left in teal closed circles), time to 50% plasmon intensity as per a logistic fit of the data (*x*_0_ left in orange triangles) and absorbance at λ = 540 nm for the purpald assay (right in purple open circles) for 6 year single cask sample series. All data sets had a water background subtracted, and measurements of triplicate samples averaged for plotting. Error bars show standard deviation in absorbance between three replicates, or for *x*_0_ the standard error of the fit. In general, high absorbance in the FC and purpald assays is expected to correlate with a short time to 50% plasmon intensity and vice versa, but this is not true in every case.

We next examined how the chemistry of the sample influences the production of Au NPs and compared our heuristic assay with some other potential colorimetric tests. In a first assessment of the samples by alternative chemical means, we used the Folin–Ciocalteu (FC) reagent as a measure of “total reductive potential” (sometimes referred to as gallic acid equivalency). This reagent reacts with phenols and non-phenolic reducing substances (particularly those found in cask-aged spirits, as well as biological reductants) to form blue chromogens (λ_max_ = 750 nm) as a result of the oxidation of the molybdenum and tungsten in the reagent.^24^ When treated with the FC reagent (Figure 3), each whisky developed a blue color in comparison to the control samples of vodka, 40% ethanol solution and water, all which remained colorless. A more intense blue product (with a higher absorbance value at λ = 750 nm) was seen in the nominally older whisky samples, where contact was made with the oak casks for longer, developing more complex congeners including aromatic aldehydes, and those which had been produced with peated malt, adding more phenolic compounds to the spirit. The FC reagent alone may be another good “quick measure” of spirit agedness; however, its emission shape cannot offer the absorbance peak shift/broadening that may be indicative of the particular congeners present that stabilize the final Au NPs formed, and this is a topic we hope to explore further.

We undertook an analysis of the congeners contained in our whisky samples that may be responsible for the formation and stabilization of Au NPs. For low volume samples (the branded samples), we used NMR, and for those with more available volume (the single cask series), the gold-standard, HPLC. In each case, it was possible to identify a range of aliphatic and aromatic aldehydes and acids, alongside sugars that are likely to reduce Au^3+^ to Au NPs (Figures S9 and S10 and Tables S4–S6). While multivariate analysis between rate of reduction (Figures S5, S6, and S11), plasmon characteristics, and congener concentration failed to identify any very strong correlations in our small sample set, there was a weak correlation with several key aldehydes (e.g., acetaldehyde, vanillin, furfural, syringaldehyde, and others), indicating that this is a functional group with the potential to reduce Au^3+^ and other metals.

To investigate this further, we tested another parallel colorimetric assay for aldehyde content. The purpald reagent can be used to detect aldehydes because, under basic conditions, the triazole chromogen condenses with aldehydes forming an unstable intermediate that can then be oxidized to a magenta-purple tetrazine product, with the observed color depending on the substituents of the aldehyde group. The test is specific and sensitive to aldehydes versus other common functional groups (e.g., ketones, esters, and amides) which do not yield a colored product.^25^ Using the 6 year single cask series, the absorbance spectra of the colored product arising from the interaction between the purpald reagent and aldehyde congeners in each whisky sample increased with the increase in sample age (Figure 3B), correlating with the HPLC data in Table S6 showing increasing aldehyde concentration with the increase in aging. In the case of the single cask whisky, we hypothesize that the dominant reducing congeners are aldehydes, given the similar trends of this sample series when comparing the FC and purpald reagents. However, there is not a direct correlation between aldehyde content and overall reducing power as measured by the FC reagent (Figure 3A), or by the Au NP assay for the different whisky brands: this suggests that many different congeners, not purely aldehydes, play a role in the formation of Au NPs in collaboration and competition with each other.

A series of spiking experiments was undertaken to see if any one congener (or combination of congeners) stood out for their reducing potential when mixed with Au^3+^ at realistic concentrations (Figures S12 and S13). An acidified 40% solution of EtOH (whisky is typically around pH 4) was spiked with reducing sugars, and with common acids and aldehydes identified in HPLC and NMR data. Acetaldehyde, furfural, gallic acid, 5-HMF, syringaldehyde, syringic acid, vanillin, and tannic acid (a group of polyphenols known to play a role in Au NP syntheses,^26^ and released from woods such as oak, albeit often as hydrolyzed gallic acid) were the congeners identified and tested.^24^ While tannic acid was not easily identified in the NMR measurements due to its polymeric nature, gallic acid was clearly found, and traces of tannic acid are likely present, having been measured in whisky previously.^27^

Single congener and mixed “cocktail” experiments highlighted that very few of these common congeners are capable of reducing or stabilizing Au^3+^ to Au NPs at this pH and these concentrations. None of these cocktails were capable of producing a strong signal in the purpald assay. An interesting observation is that the sugar content does not seem to be particularly critical to reduction by whisky (unlike in soft drinks),^22^ and this implies that caramel addition (a legally allowed colourant in Scotch whisky) should not adversely impact the test results, as also evidenced by the Bowmore sample (Figure S2).

Only gallic acid and tannic acid produced plasmonically active Au NPs, and tannic acid was clearly highlighted as a potential reductant and capping agent, generating an obvious and strong Au NP signal. Given that the extraction process of tannins from the casks and the breakdown of these to gallic acid and related acids and aldehydes is the “aging process” of spirit to make whisky, it is therefore possible to relate the rate of congener extraction from the cask wood into the spirit to Au NP formation and hence link Au NP formation to agedness. However, if tannic acid was the only reducing agent active in the samples, then the diversity of shapes and sizes and therefore plasmon band shape of the Au NPs produced would not be seen, and so we are continuing to investigate the potential identities of species that contribute to our Au NP whisky agedness assay.

## Conclusions

We have shown that whisky is capable of producing and stabilizing Au NPs in a rapid reaction. We posit that this is due to flavor and color compounds (congeners) in the whisky that are extracted from or generated in the casks during aging (maturation) and that rapid production of Au NPs correlates with increased spirit maturity, making this a portable and applicable, on-site test.

Our test could be useful for monitoring the maturation of casks in a distillery warehouse. We have shown that there is a link between reductive potential, maturation length, and congener concentration with a 6 year old single-cask sample series. We have also attempted to identify the key congeners in whisky that lead to the formation and stabilization of the Au NPs to help perfect this assay as a “fingerprinting” tool for analyzing cask samples using rate of plasmon generation, plasmon position, and peak shape. The mechanism of Au NP formation is non-trivial but our experiments suggest that particle nucleation and growth seems to be “classical”, with growth curves fitting well to the “Finke–Watzky model” (slow continuous nucleation and autocatalytic growth).^28^ Carbonyl species in the sample contribute to the reduction,^29^ evidenced by the purpald assay, and tannic acid and related phenolic species also contribute (evidenced by the spiking experiments and FC tests). However, each factor will be in balance in each sample based on the congener concentrations. Compared to the commercially available FC or purpald chromogenic reagents, our test provides additional layers of information, and, due to the sensitive nature of plasmonic NPs, this reductive test shows larger changes in relation to congeners present (as seen by extinction profile and colloidal color differences).

The data presented for both commercial and single cask whisky reduced Au NPs demonstrate the broad applicability of the technique, and the rapid and inexpensive nature of the test shows promise for use on the floor in maturation warehouses. In our study, a bench-top plate reader was used to enable high-throughput measurements on multiple samples; however, a portable UV–visible spectrometer for measuring a few samples at once could be simply developed based on a smartphone or other similar technology.^30^ The test is inexpensive, with the value of the small 50 μL sample of spirit estimated to be greater than that of the gold reagent, and the whole process costing a penny or less (calculation in the Supporting Information).

Having shown the appearance of a plasmon band and the rate of metal reduction to Au NPs can be linked to spirit agedness, we are now focusing on developing a better understanding of the extinction profiles of the Au NPs to try to relate these to congener concentrations and even flavor notes. We hope to augment the test in the future to be able to also detect higher alcohols and sugars, and by comparing these correlations with the opinion of a master blender, we aim to connect the chemical and sensory definition of whisky agedness.

## Supporting Information Available

Detailed materials and methods data, additional UV–visible extinction spectra for samples described in the text, ^1^H NMR spectra and HPLC data for congener quantitation in select samples, and kinetic fits of plasmon growth (PDF)
