# Supplementary material for: Growth of Plasmonic Nanoparticles for Aging Cask-Matured Whisky
Source: ACS Appl Nano Mater. 2022 Oct 6;5(10):15362–8. doi: 10.1021/acsanm.2c03406 (PMC9624259; doi:10.1021/acsanm.2c03406)
Supplement: Supplementary file 1 — an2c03406_si_001.pdf [file an2c03406_si_001.pdf]

Supporting Information

Growth of Plasmonic Nanoparticles for Aging Cask-Matured Whisky

Jennifer Gracie[1], Francesco Zamberlan[1], Iain B. Andrews[2], Brian O. Smith[3], William J. Peveler[1]*

[1] School of Chemistry, University of Glasgow, Glasgow G12 8QQ, United Kingdom
* Email: william.peveler@glasgow.ac.uk
[2] The Scotch Whisky Research Institute, Edinburgh EH14 4AP, United Kingdom
[3] School of Molecular Biosciences, University of Glasgow, Glasgow G12 8QQ, United Kingdom

Materials & Methods

Materials

Hydrogen tetrachloroaurate (III) trihydrate (99.99%) and silver nitrate (99.9%) were supplied by Alfa Aesar. Purpald (≥99%) reagent, Folin-Ciocalteu reagent, tannic acid, sodium hydroxide (NaOH), sodium periodate ($NaIO_4$) and ethanol (EtOH) were purchased from Merck. Sodium carbonate (Na_2CO_3) was from Fisher Scientific. Deuterated solvents were purchased from Goss Scientific. All chemicals were used without further purification. Branded whisky samples were shop-bought, and the 6-year single cask samples were kindly supplied by the Scotch Whisky Research Institute. All aqueous solutions were prepared using deionised water (15 MΩ). Costar® 96-well plates (half area, clear polystyrene) were used for reactions and extinction measurements. Holey carbon film on copper grids (300 mesh) were purchased from EM Resolutions.

Instrumentation

A Tecan microplate reader (Spark®) was used to collect extinction and absorbance data. Nanoparticles were imaged using a JEOL 1200 EX TEM running at 80kV, with digital Images (tifs) captured using a Cantega 2k x 2k camera and Olympus ITEM Software. White light photographs were obtained using a Samsung S8 smartphone. NMR spectra were obtained using methods detailed below on a Bruker Avance III 500 MHz instrument, with a Bruker 5 mm QXI probe. Spectra were processed in TopSpin 3.6.2 and MNova 14 for peak picking and normalisation. HPLC-MS data were provided directly by the Scotch Whisky Research Institute.

General Au NP Synthesis

In a typical experiment, 50 µL of neat whisky was added to a well in a 96-well plate followed by 50 µL of an aqueous 0.25 mM Au^{3+} solution, giving a final Au^{3+} concentration of 0.125 mM. All whiskies and controls (water, vodka, 40% EtOH solution) were added to the wells before adding the Au^{3+} solution using a multichannel pipette and beginning kinetic measurements immediately. Kinetics were followed for up to 2 hours.

A similar method was used for the formation of Ag NPs, using 50 µL of an aqueous 32 mM Ag^+ solution, resulting in a final Ag^+ concentration of 16 mM in each sample. Kinetics of particle growth were followed over three days.

Commercial whiskies investigated

Table S1 - Whisky Brands used to form AuNPs.

Brand	Whisky Type	Place of Origin	Maturation Cask Type	Notes
Tesco Special Reserve Whisky	Blended malt and grain	Highlands, Scotland	Oak, likely bourbon	Minimum 3-year maturation, likely young
Chita	Single grain	Japan	Bourbon, sherry and wine	43% ABV
Jameson	Blended grain	Ireland	Bourbon and sherry	
Jura 10	Single malt	Jura, Scotland	Bourbon and sherry	Peated
Highland Park 12	Single malt	Orkney, Scotland	Sherry	Peated
Laphroaig Select	Single malt	Islay, Scotland	Quarter Casks, Bourbon, sherry and new oak	Peated
Bells	Blended malt and grain	Scotland	Unspecified	
Ben Riach 12 y/o	Single malt	Speyside, Scotland	Bourbon, port and sherry	
Ben Riach 16 y/o	Single malt	Speyside, Scotland	Bourbon, port and sherry	
Ben Riach 20 y/o	Single malt	Speyside, Scotland	Bourbon, port and sherry	
Bowmore 12 y/o	Single malt	Islay, Scotland	Unspecified	Peated, caramel colour added
Bulleit Bourbon	Barley, rye, and corn	Kentucky, USA	Virgin Oak	
Glenfiddich (Fire & Cane)	Single malt	Speyside, Scotland	Bourbon and rum	
Glenmorangie 10 y/o	Single malt	Highlands, Scotland	Bourbon	
Woodford Reserve, Distiller's select	Barley and rye	Kentucky, USA	Virgin Oak	43.2% ABV
Absolut Vodka	Winter wheat	Sweden	Not Cask Matured	

Purpald Reagent Method

Scaled protocol from Quesenberry *et al.*[1] Stock solutions were prepared fresh prior to each experiment: 34 mM Purpald solution (25 mg in 5 mL 2 M NaOH) and 33 mM $NaIO_4$ solution (35 mg in 5 mL 0.2 M NaOH). Into each well 30 µL of neat whisky was added, followed by 30 µL Purpald solution and shaken briefly in the plate reader before incubating inside the instrument for 20 minutes. 30 µL of $NaIO_4$ solution was then added, the plate was briefly shaken to mix reagents, and absorbance scans were measured.

Folin-Ciocalteu Reagent Method

Scaled protocol from Chetrariu *et al.* [2] Into each well 5 µL of neat whisky was added, followed by 50 µL of Folin-Ciocalteu reagent (diluted 10x in water); this mixture was left for 5 minutes before 45 µL of Na_2CO_3 (7.5% w/v, 0.375g in 5 mL water) was added. The plate was briefly shaken, and absorbance scans collected after a 30-minute incubation.

Tannic Acid Spiked EtOH

Concentration range of tannins in whisky was previously identified by Leibmann *et al.* [3] The range tested was corrected for converting US Proof to UK ABV alcohol content of whisky. Tannic acid standards were prepared in 40% EtOH at pH 4 (4 mL EtOH + 6 mL water, pH corrected by adding 10 µL 6M HCl) solution at 0.1, 0.2, 0.3, 0.4 and 0.5 mg/mL. 50 µL of tannic acid solution was added to a well before adding 50 µL of 0.25 mM Au^{3+} solution. Extinction spectra were collected after incubating the plate for 1 hour.

Additional data on Au NP formation and sample whiskies

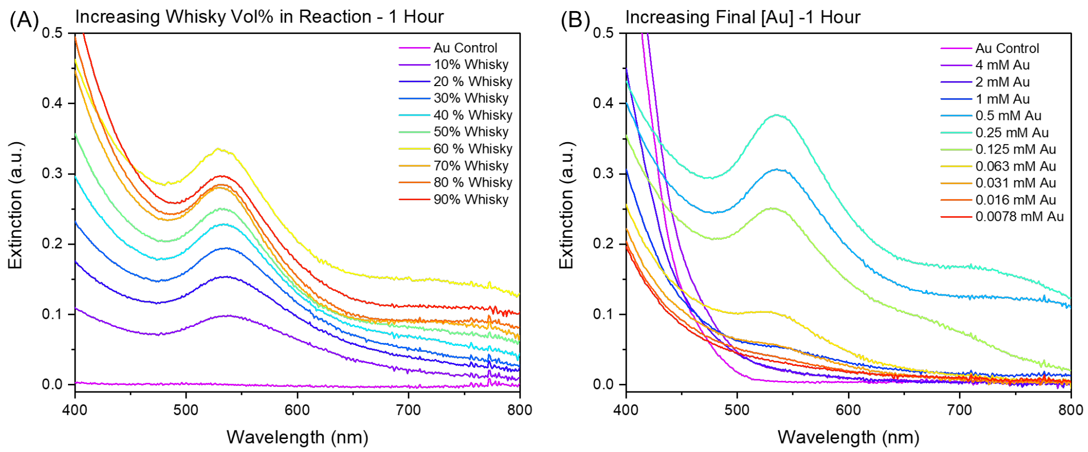

Figure S1 - Extinction spectra for reaction optimisation using the Tesco whisky sample. (A) Increasing total whisky volume in reaction, keeping Au^{3+} concentration (0.125 mM) and volume (100 µL) constant. (B) Increasing final gold concentration in sample from 0.0078-4 mM, keeping whisky volume constant. Background water reference was subtracted in each case.

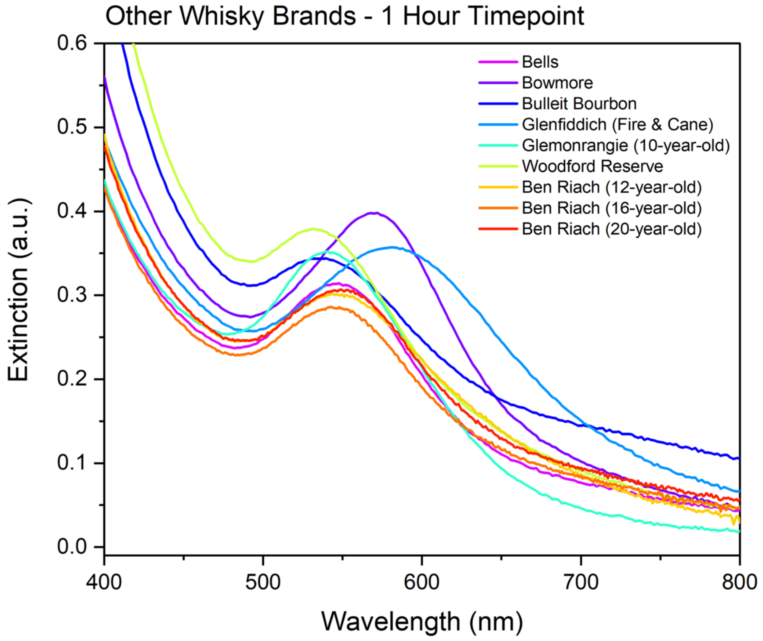

Figure S2 - Extinction spectra of Au NP formation after 1 hour, reduced by various whisky brands. Background water reference subtracted.

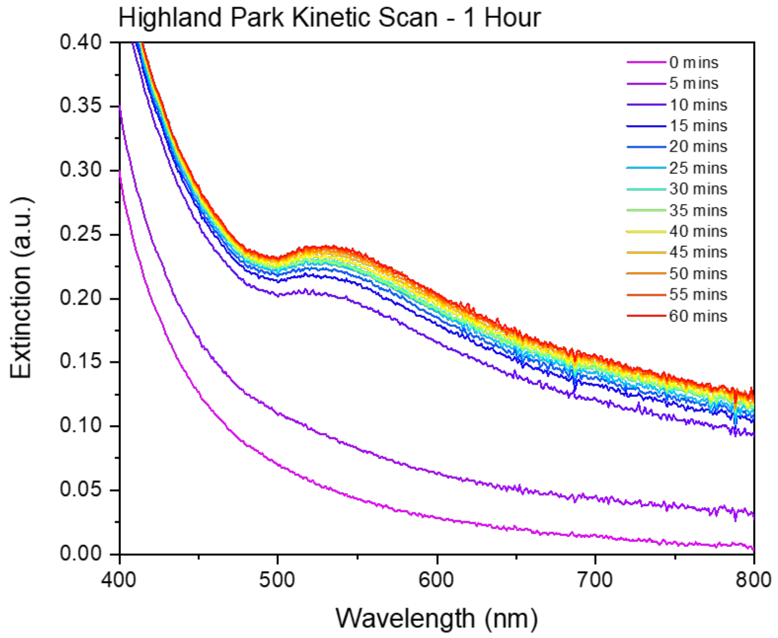

Figure S3 - Extinction spectra of Au NP formation over 1 hour kinetics scan using Highland Park whisky. Triplicate samples scanned at 5 minutes intervals, background water reference subtracted, and replicates averaged prior to plotting.

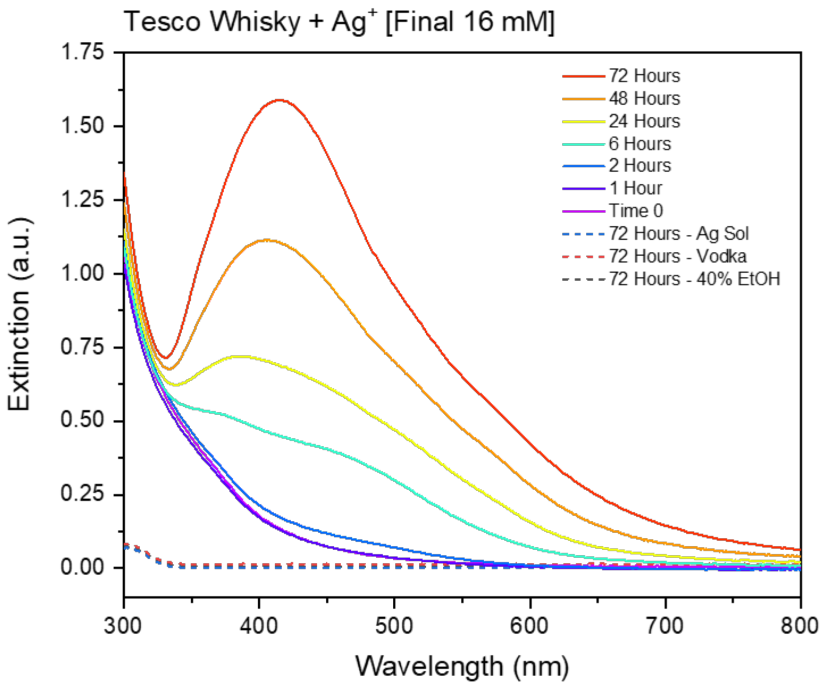

Figure S4 - Extinction spectra of Ag NP formation over 3 days. 50 µL of 32 mM Ag^+ solution + 50 µL neat Tesco Whisky. Background water reference subtracted. Dashed lines represent control samples: Ag^+ solution + water, Ag^+ + neat Vodka, Ag^+ + 40% EtOH solution.

Kinetic fits for Au NP growth

The initial plasmon growth was modelled with a logistic growth model, over a 25-minute time period, to fit the initial condition, S-shaped growth curve and the initial portion of the plateau.

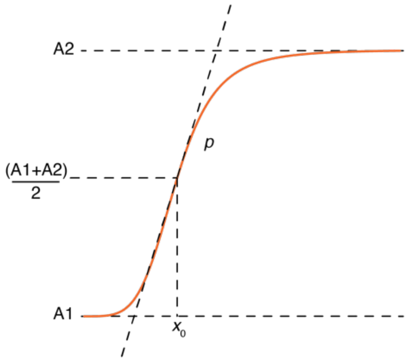

$$y = \frac{A_1 - A_2}{1 + \left(\frac{x}{x_0}\right)^p} + A_2$$

Where *A1* is the initial absorption, *A2* the final absorption, x_0 is the time to half-maximum intensity, and *p* is proportional to the steepness of the centre of the curve.

As the particles settled over time in some cases, fitting longer time frames added no benefit and in some cases was deleterious to the quality of fit. In other cases, the growth was already started at the first time point, or a secondary slow ripening occurred, and so the initial portion of the curve fit was less representative of the whole growth curve. However, in most cases x_0

was a reasonable value to extract and correlated well with the data points, so this was used as a proxy for initial rate of particle growth by reduction of the gold salt.

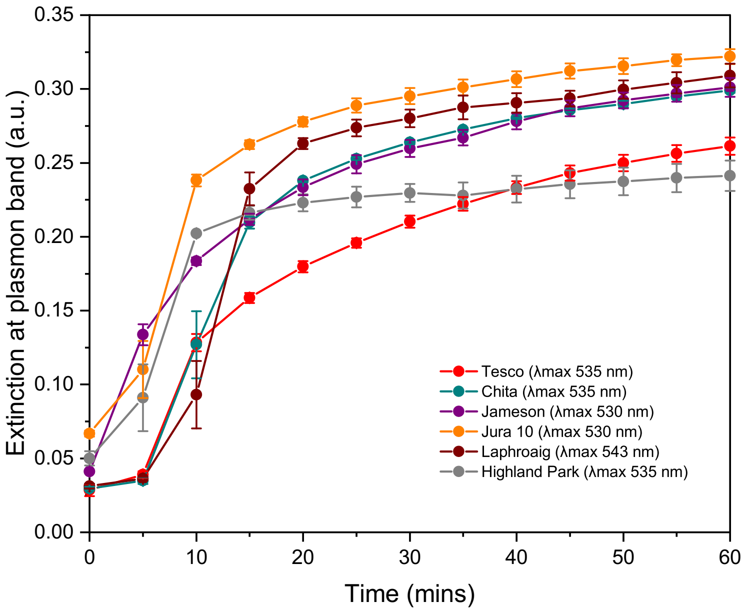

Figure S5 – Kinetic data for whisky brands over 1 hour (average of three replicates ± 1 SD)

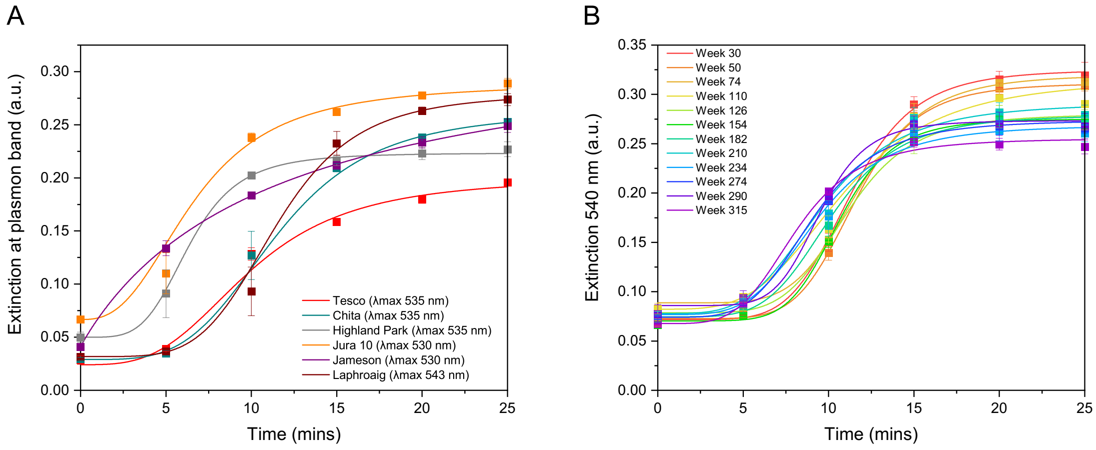

Figure S6 – Kinetic data for whisky brands (A) and single cask (B) over 25 minutes, with fit shown against the average of three replicates ± 1 SD.

Table S2 – Tabulated fit data for branded whiskies and single cask sample

	A1		A2		x0		p		Fit	
Brands (λ_{max} of plasmon)	Value	Std. Error	Value	Std. Error	Value	Std. Error	Value	Std. Error	Red. X^2	Adj. R^2
Tesco (535 nm)	0.024	0.010	0.200	0.013	9.919	1.067	3.245	0.818	6.864	0.987
Chita (535 nm)	0.029	0.002	0.262	0.004	11.464	0.494	3.988	0.466	3.127	0.999
Highland Park (535 nm)	0.050	0.003	0.223	0.003	6.368	0.392	4.359	0.670	0.398	0.998
Jura 10 (530 nm)	0.067	0.004	0.289	0.012	6.628	0.804	2.674	0.999	3.353	0.997
Jameson (530 nm)	0.041	0.0002	0.346	0.021	11.541	1.714	0.971	0.078	0.074	1.000
Laphroaig (543 nm)	0.032	0.001	0.279	0.006	11.468	0.486	4.852	0.439	0.718	0.999
Single Cask (540 nm)										
Week 30	0.072	0.004	0.325	0.016	11.290	0.579	5.972	1.540	2.729	0.992
Week 50	0.073	0.003	0.311	0.005	11.563	0.309	6.431	0.926	1.004	0.998
Week 74	0.089	0.004	0.320	0.005	11.792	0.220	5.937	0.502	1.595	0.998
Week 110	0.082	0.001	0.316	0.020	10.832	1.215	3.739	0.483	2.262	0.992
Week 126	0.079	0.004	0.280	0.010	11.062	0.422	5.746	1.482	0.873	0.993
Week 154	0.070	0.003	0.275	0.006	10.647	0.255	6.810	1.197	1.472	0.997
Week 182	0.075	0.004	0.279	0.011	10.183	0.480	5.031	1.307	0.867	0.993
Week 210	0.078	0.003	0.291	0.013	9.587	0.812	4.074	0.521	1.907	0.991
Week 234	0.077	0.005	0.269	0.010	9.299	0.726	4.382	0.812	1.490	0.991
Week 274	0.074	0.004	0.274	0.008	9.058	0.442	4.581	0.826	5.262	0.994
Week 290	0.086	0.007	0.273	0.009	9.492	0.326	6.946	3.760	0.916	0.991
Week 315	0.068	0.003	0.255	0.006	8.089	0.227	4.348	0.288	1.921	0.998

Analysis of TEM data

Particle counting and sizing analysis of the Au NPs in the TEM images was performed using the tools in the Image J package. Images were scaled and thresholded to create black particles on a white background, and the watershed function was used to separate conjoined particles in the image. The particles were then fitted with ellipses, with a minimum area threshold to exclude particles that had been over-divided with the watershed function, or in some cases, where image speckle remained.

Table S3 – Measured properties and notes from TEM images

Sample	Mean Size[a]	Median	N	Range[b]	Anisotropy[c]	Notes
Tesco	14.9 ± 7.2 nm	15.6 nm	565	48.1 – 1.0 nm	1.26 ± 0.34	Largely spheroidal, a population of a few larger particles, some small triangles, trapezoids etc.
Chita	27.1 ± 10.5 nm	27.9 nm	280	65.7 – 4.0 nm	1.22 ± 0.17	Larger edged/faceted particles with obvious vertices
Jameson	14.0 ± 4.0 nm	13.6 nm	155	33.2 – 4.1 nm	1.31 ± 0.3	Largely spheroidal, no large particles or other shapes
Jura 10	19.25 ± 16.7 nm	15.8 nm	133	110 – 1.8 nm	1.27 ± 0.25	Largely small and spheroidal but a few much larger particles
Highland Park	78.1 ± 33.0 nm	71.9 nm	186	195 – 18.4 nm	1.25 ± 0.25	Large (> 50 nm) spheroidal clusters of NPs typically < 20 nm

a) Mean size on major axis of fitted ellipse ± standard deviation
b) Maximum and minimum dimensions of fitted ellipses
c) The average ratio of major and minor dimensions of ellipses ± standard deviation as a measure of circularity

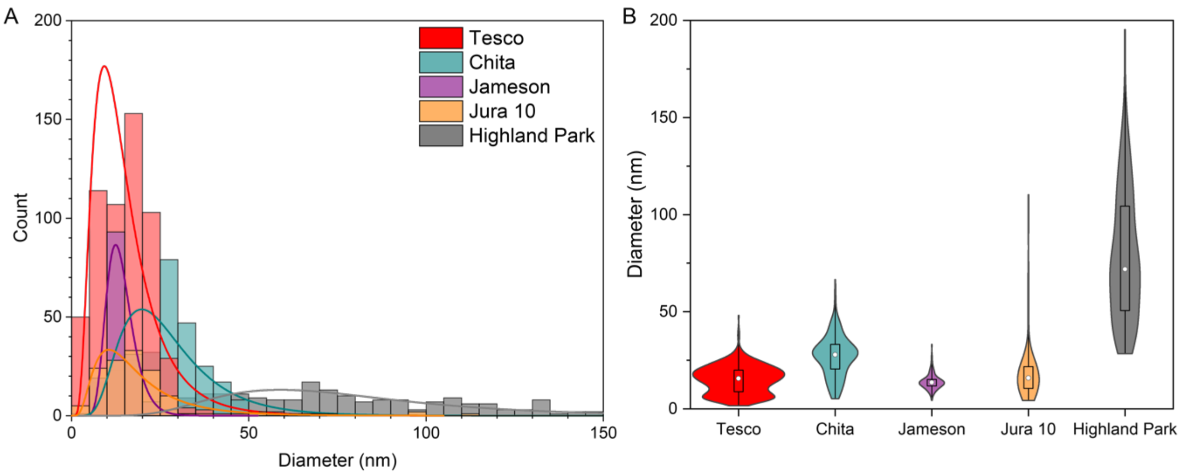

Figure S7 – (A) Histogram of particle sizes for the five samples imaged, with fitted log-normal functions. The peak of the curve corresponds to the sample mode. (B) Violin plots for the measured particles showing distribution, range (vertical line), interquartile range (box) and median (white circle). The small number of large particles in the Jura 10 sample that skew the mean is notable.

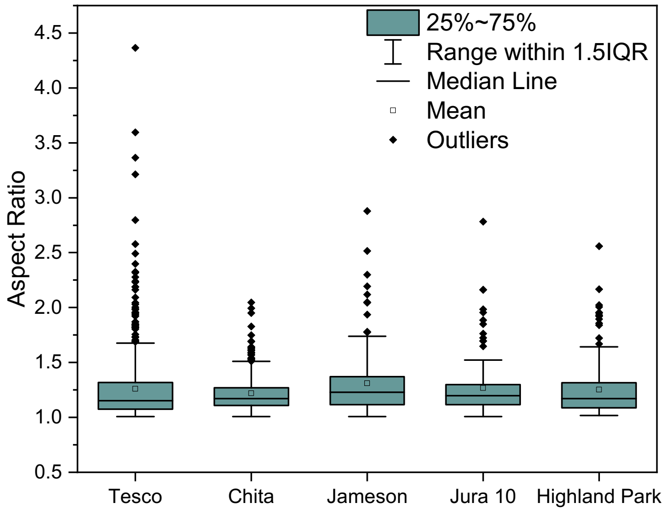

Figure S8 – Box and whisker plot of the aspect ratio (ellipse maximum divided by ellipse minimum) for each particle. The irregularity in shape of the Tesco sample Au NPs is notable.

Investigating congeners responsible for whisky reduction potential

The whisky sample were prepared in high precision NMR tubes (Wilmad 535 PP7), by mixing 100 µL of buffer and 500 µL of whisky sample. The buffer for these experiments is deuterated sodium acetate/acetic acid in deuterated water containing 6 mM deuterated 3-(trimethylsilyl)-1-propanesulfonic acid sodium salt (DSS-d6) as in internal standard – see below. The final concentration in the NMR tube, as reported by Kew et al., is 1 mM for DSS-d6 and 25 mM buffer.[4]

Stock deuterated sodium acetate/acetic acid buffer at 150 mM concentration for dilution was prepared as follows: 20 mL of D_2O were mixed with deuterated sodium acetate/acetic acid and DSS-d6 (1.5 mmol each: 127.9 mg NaOAc-d3, 86 µL AcOH-d4, and 27.4 mg of DSS-d6).

Spectra were collected using the 4-step experiment described by Kew et al.[4] with scripts provided on GitHub at https://github.com/wkew/NMRScripts/ and implemented on our own spectrometer. The DSS internal standard was used to set 0 ppm and integrated to 9 protons for quantitative analysis, and assignments of chemistries were made informed by the work by Kew et al. A fuller description of the protocols and assignments are provided by the original authors.[4–6]

Figure S9 – NMR spectra of primary whisky samples for the purpose of congener content identification. All spectra are normalised to 9 protons at 0 ppm. (A) Regions of interest between 10 and 5.1 ppm (aromatics, aldehydes and acids from cask aging); (B) 4.7 and 2.8 ppm (sugars); (C) and 2.8 and 0 ppm (higher alcohols) are expanded and zoomed.

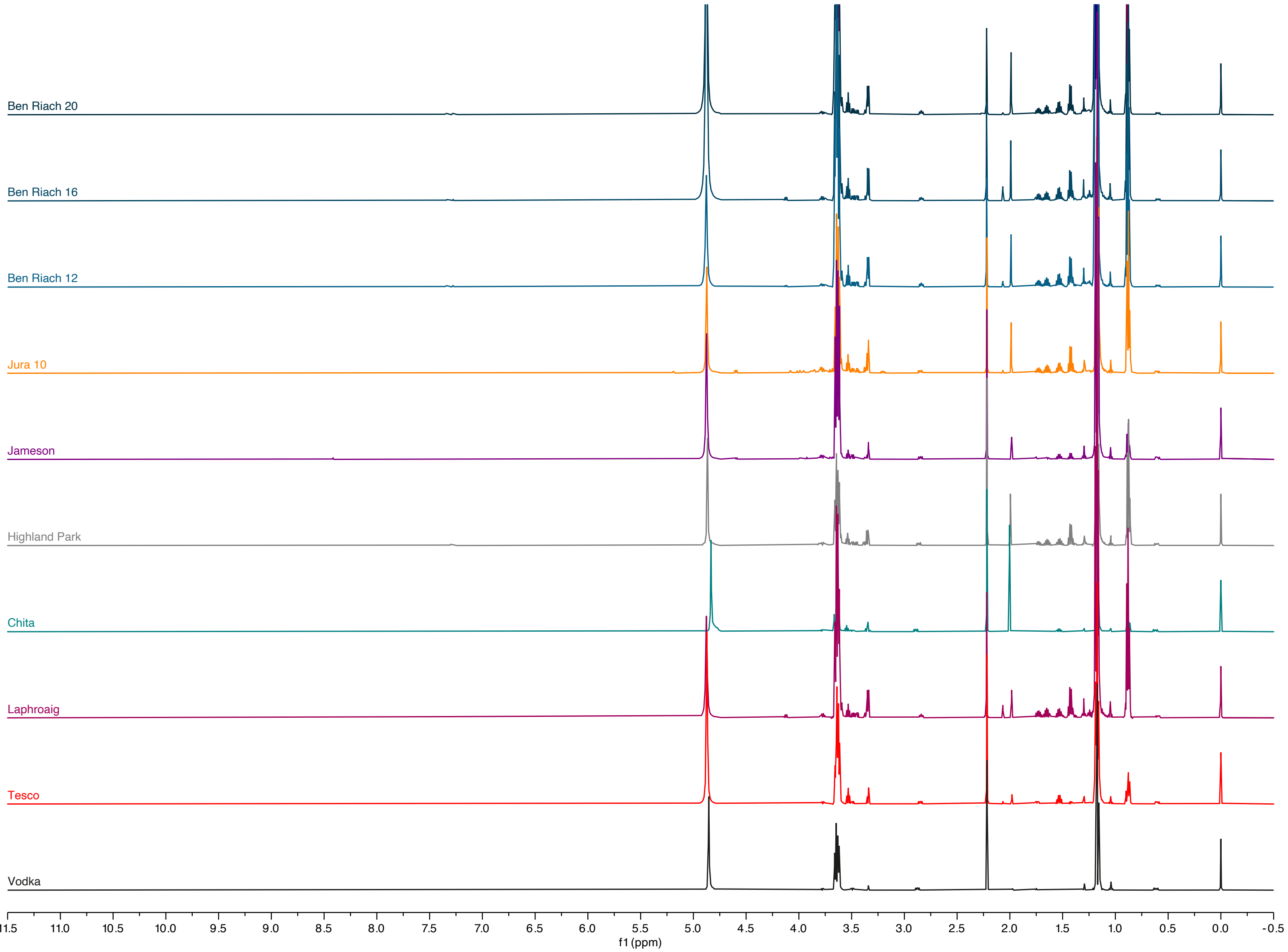

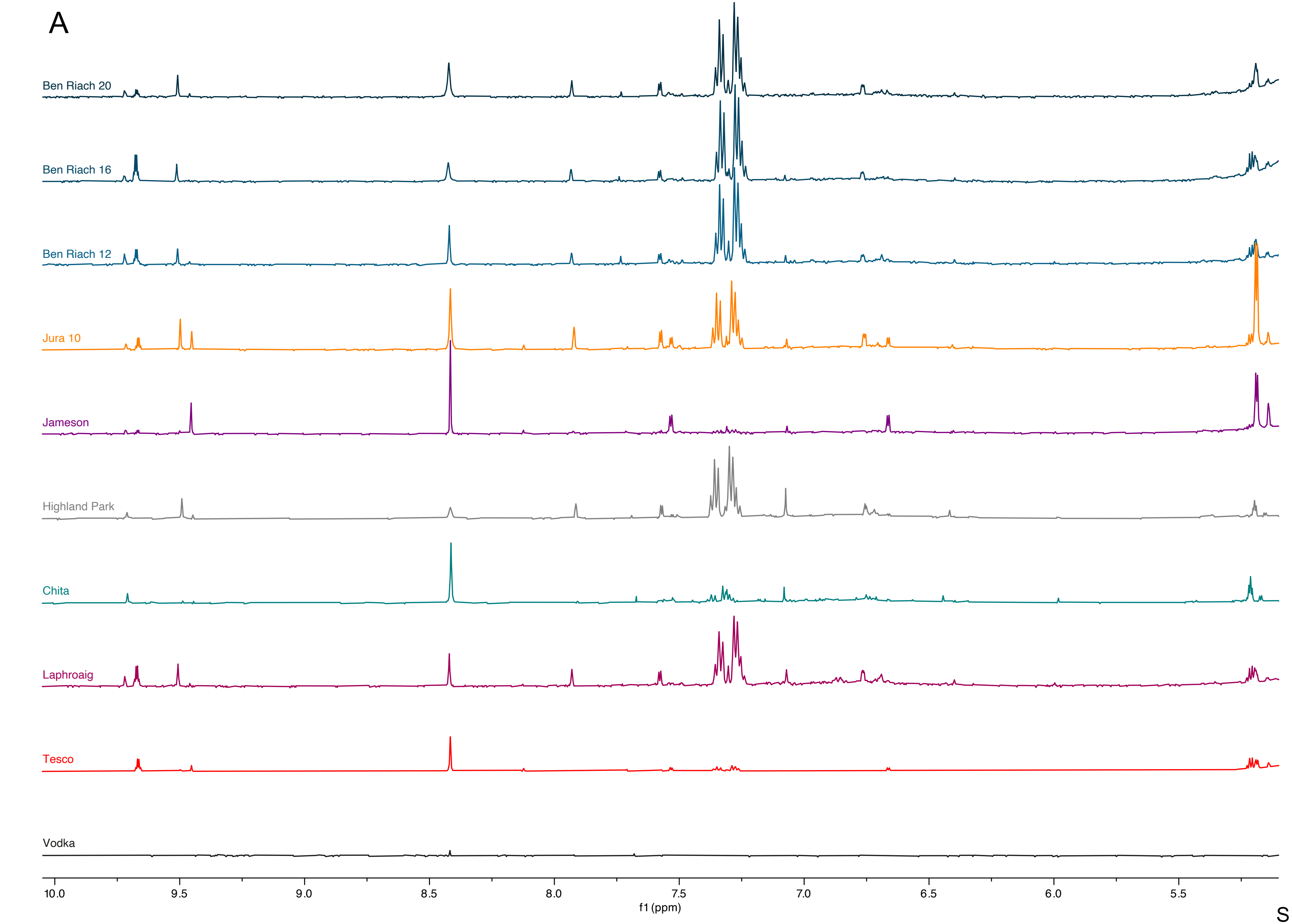
A
Ben Riach 20
Ben Riach 16
Ben Riach 12
Jura 10
Jameson
Highland Park
Chita
Laphroaig
Tesco
Vodka
10.0
9.5
9.0
8.5
8.0
7.5
7.0
6.5
6.0
5.5
f1 (ppm)

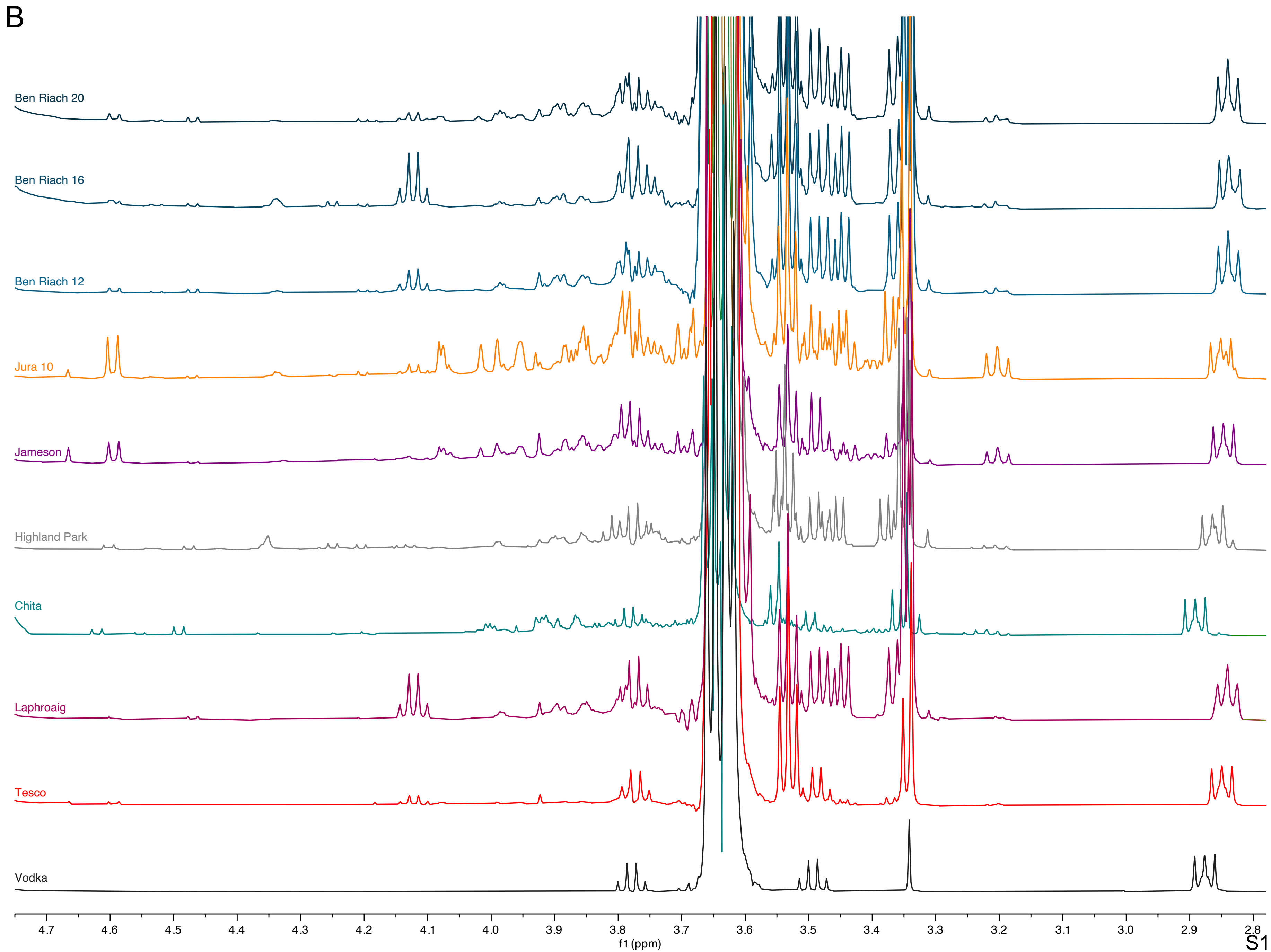
B
Ben Riach 20
Ben Riach 16
Ben Riach 12
Jura 10
Jameson
Highland Park
Chita
Laphroaig
Tesco
Vodka
4.7
4.6
4.5
4.4
4.3
4.2
4.1
4.0
3.9
3.8
3.7
3.6
3.5
3.4
3.3
3.2
3.1
3.0
2.9
2.8
f1 (ppm)

C

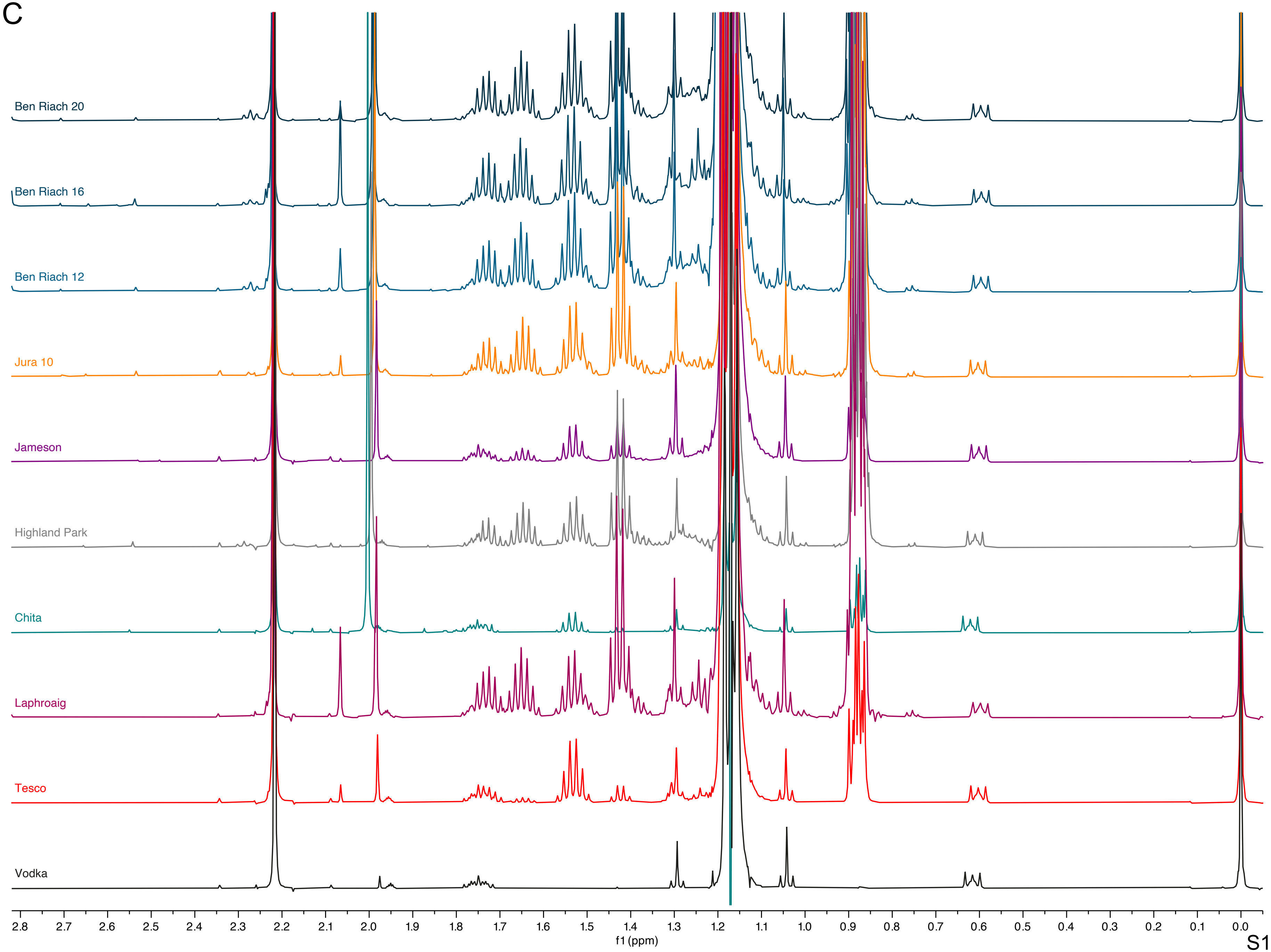

Ben Riach 20
Ben Riach 16
Ben Riach 12
Jura 10
Jameson
Highland Park
Chita
Laphroaig
Tesco
Vodka
2.8 2.7 2.6 2.5 2.4 2.3 2.2 2.1 2.0 1.9 1.8 1.7 1.6 1.5 1.4 1.3 1.2 1.1 1.0 0.9 0.8 0.7 0.6 0.5 0.4 0.3 0.2 0.1 0.0
f1 (ppm)

Figure S10 – Example NMR spectra (Jura 10) with key congeners identified

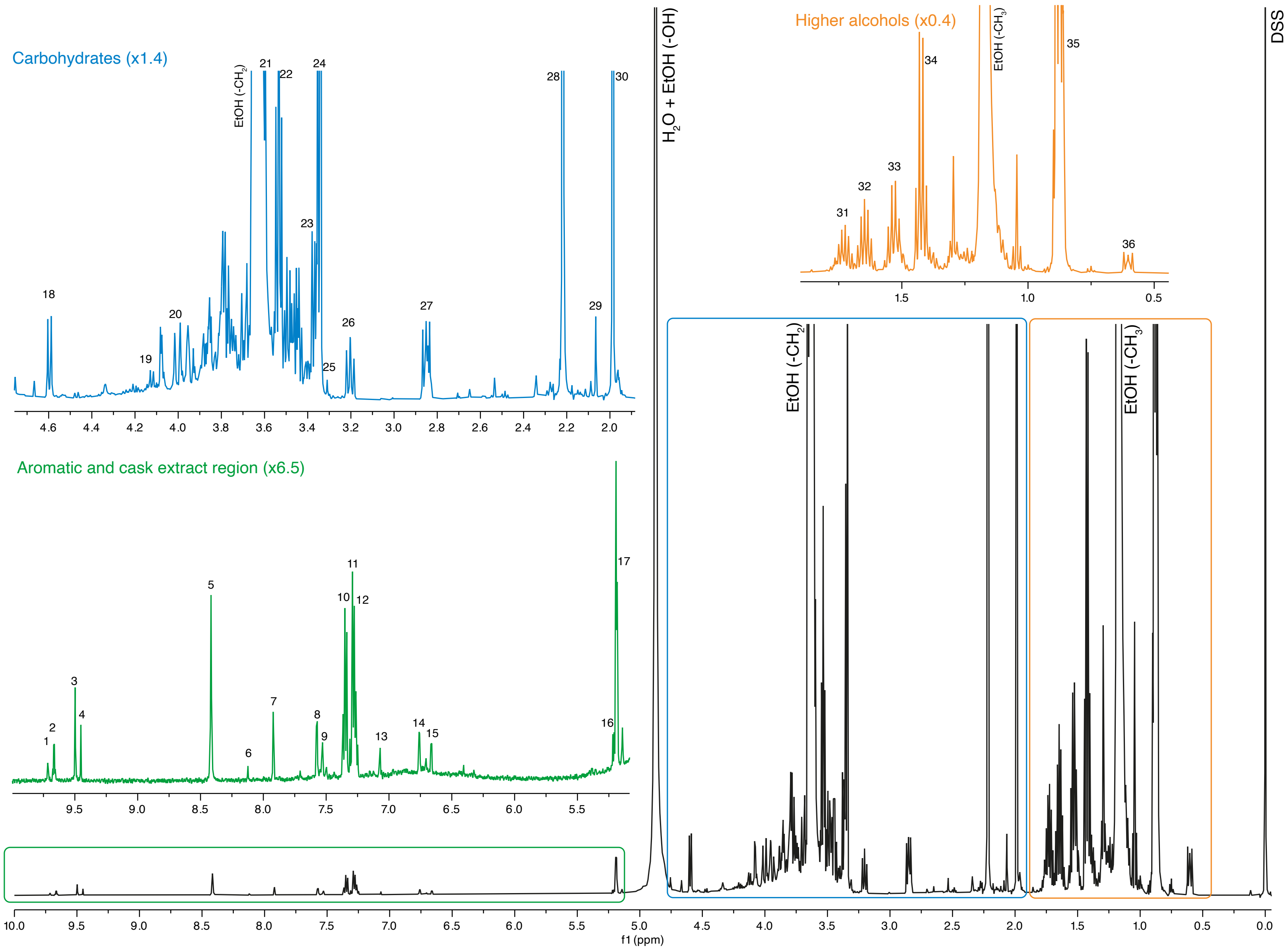

Table S4 – List of whisky congeners identified in NMR spectra based on Kew et al. Uncertain IDs are marked with a suggestion and/or a '?'.

1	Syringaldehyde	19	Ethyl acetate
2	Acetaldehyde	20	β-D-fructopyranose
3	Furfural	21	3-Methylbutanol
4	5-HMF	22	*n*-Propanol
5	Formic acid	23	2-Methylbutanol
6	Ethyl formate	24	Isobutanol
7	Furfural	25	Methanol
8	Furfural	26	Glucose?
9	5-HMF	27	2-Phenylethanol + ?
10	2-Phenylethanol	28	Acetaldehyde + ?
11	Syringaldehyde	29	Ethyl Acetate
12	2-Phenylethanol	30	Acetate/acetic acid
13	Gallic acid	31	Isobutanol
14	Furfural	32	3-Methylbutanol
15	5-HMF	33	*n*-Propanol
16	Acetaldehyde ethyl hemiacetal	34	3-Methylbutanol
17	α-D-glucopyranose	35	Other alcohol methyl groups
18	β-D-glucopyranose	36	?

Phenolics from peat are identified as the largely broad background peaks under the region 6.5 – 7 ppm. This is most pronounced in the Jura, Laphroaig and Highland Park samples. As confirmed in previous work, 3-methyl butanol is more prevalent in single malts than blends, and 2-phenylethanol is low in the grain whiskies/shorter matured whiskies.

Table S5 – Tabulated data for congeners detected in samples analysed by NMR. Values in mM. A '–' indicates value less than noise threshold. *Peak for syringic acid has some overlap with 2-phenylethanol making the integrals less accurate.

	ppm	no. Protons	Vodka	Tesco	Laphroaig	Chita	Highland Park	Jameson	Jura	Ben Riach 12	Ben Riach 16	Ben Riach 20
DSS	0	9	1	1	1	1	1	1	1	1	1	1
Acetaldehyde	9.68	1	–	0.108	0.159	–	–	0.045	0.111	0.154	0.223	0.083
Furfural	9.51	1	–	0.014	0.095	–	0.120	0.019	0.131	0.083	0.087	0.098
Formic acid	8.42	1	0.023	0.144	0.151	0.375	0.159	0.306	0.453	0.221	0.213	0.319
Syringic acid*	7.32*	2	–	0.006	0.129	0.052	0.056	–	0.120	0.174	0.197	0.156
Gallic acid	7.10	2	–	–	0.075	0.039	0.075	0.018	0.026	0.026	0.020	–
5-HMF	9.47	1	–	0.025	0.019	–	0.023	0.125	0.074	0.036	0.000	0.032
Syringaldehyde	9.72	1	–	0.000	0.050	0.017	0.059	0.034	0.047	0.095	0.059	0.062

Table S6 – Tabulated data for congeners detected in single cask sample over time, by HPLC-MS (data provided by Scotch Whisky Research Institute, adjusted to mM from ppm and corrected for dilution to 40% ABV as used in the experiments).

	Sampling week:												
	0	30	50	74	110	126	154	182	210	234	274	290	315
Gallic Acid	0.000	0.021	0.024	0.030	0.034	0.037	0.040	0.038	0.037	0.043	0.047	0.046	0.049
Ellagic Acid	0.000	0.027	0.029	0.034	0.039	0.040	0.042	0.042	0.043	0.045	0.046	0.047	0.047
Coniferaldehyde	0.000	0.009	0.009	0.010	0.011	0.011	0.012	0.011	0.012	0.012	0.012	0.013	0.013
Vanillin	0.000	0.008	0.010	0.013	0.015	0.016	0.018	0.019	0.020	0.022	0.023	0.024	0.024
Vanillic Acid	0.000	0.002	0.003	0.003	0.004	0.004	0.004	0.005	0.005	0.006	0.006	0.006	0.007
Sinapaldehyde	0.000	0.018	0.018	0.018	0.018	0.018	0.018	0.016	0.016	0.016	0.016	0.015	0.016
Syringaldehyde	0.000	0.025	0.027	0.033	0.037	0.039	0.042	0.045	0.045	0.047	0.050	0.051	0.052
Syringic Acid	0.000	0.004	0.004	0.005	0.006	0.006	0.007	0.008	0.007	0.008	0.009	0.009	0.010
5-HMF	0.000	0.013	0.013	0.014	0.014	0.014	0.015	0.015	0.015	0.016	0.016	0.015	0.017
Acetaldehyde	0.024	0.121	0.122	0.157	0.171	0.203	0.231	0.210	0.223	0.254	0.233	0.275	0.323
Ethyl Acetate	0.763	1.741	1.996	2.275	2.458	2.969	3.047	2.842	3.154	3.445	3.345	3.586	3.835
Acetal	0.014	0.133	0.144	0.162	0.162	0.196	0.199	0.177	0.191	0.207	0.197	0.214	0.192
Methanol	0.505	0.617	0.648	0.690	0.683	0.803	0.798	0.728	0.779	0.841	0.785	0.832	0.901
n-Propanol	1.586	2.227	2.299	2.306	2.231	2.507	2.510	2.259	2.433	2.546	2.399	2.529	2.828
Isobutanol	1.890	3.227	3.334	3.366	3.246	3.665	3.664	3.303	3.572	3.731	3.541	3.711	4.275
2-Methyl butanol	0.031	2.024	2.072	2.104	2.020	2.268	2.296	2.067	2.239	2.330	2.200	2.318	2.407
3-Methyl butanol	0.089	5.285	5.390	5.474	5.276	5.905	5.971	5.399	5.827	6.071	5.743	6.067	6.489
Furfural	0.000	0.077	0.076	0.080	0.081	0.089	0.091	0.091	0.090	0.094	0.086	0.095	0.088

Data provided by SWRI for the cask samples, from HPLC, is more detailed than analysis possible with NMR, but concentrations of congeners are largely in the same range, and increase with increasing wood contact time over 6 years. This particular whisky appears to be higher in acetaldehyde than the other brands measured with NMR.

Hierarchical clustering analysis

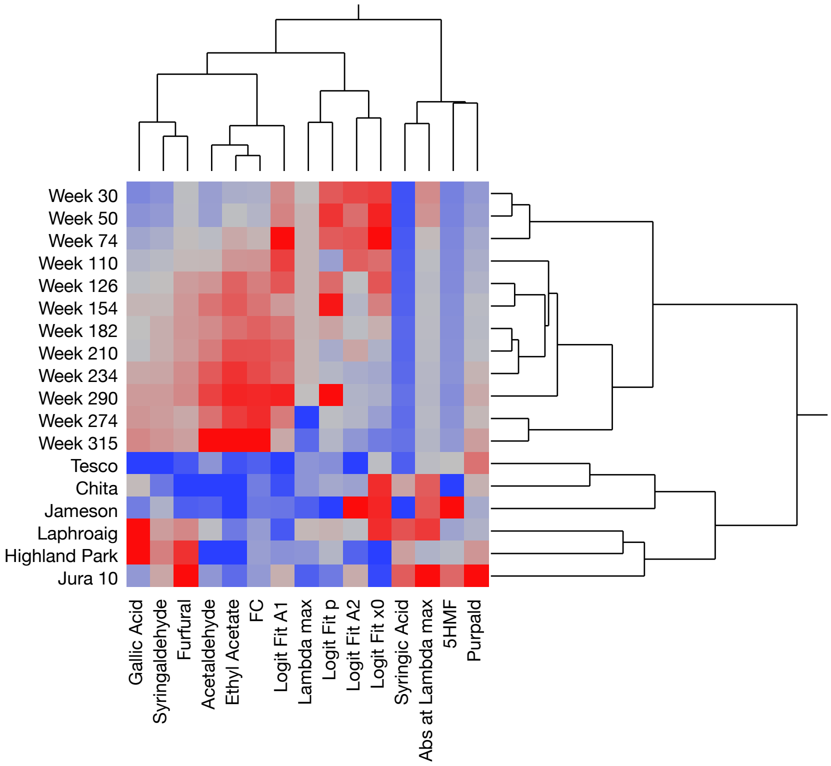

Figure S11 – Hierarchical Clustering Analysis (Ward method, performed using JMP) of samples and coincidental chemical measures by NMR, HPLC and optical analysis by the Au NP, Purpald and FC assays. Red indicates higher values and blue lower values. The single cask sample naturally clusters, as expected, and two major clusters within the brands are evident, consisting of more aged single malts, and young blends respectively. Within the measured variables, absorption at lambda max clusters with syringic acid and 5-HMF, and with the purpald assay. The other chemistries cluster with the FC assay, and the final lambda max clusters with the rate measurements, suggesting some possible covariance within these samples, but a different trend in the Au NP assays to either the FC or Purpald assays, demonstrating added value.

Creating mimic mixtures to investigate congener influence

The reduction of Au salts by single congeners at relevant concentrations was attempted. Single congeners (acetaldehyde, furfural, gallic acid, 5-HMF, syringaldehyde, syringic acid, vanillin) were prepared in a 40% EtOH (pH 4) solution at the highest concentration detected across all the whisky samples (via NMR/HPLC). These were then evaluated for reducing potential using the same conditions as described for the whiskies above (Figure S12A). In addition, ‘cocktails’ of the congeners identified were mixed at the concentrations detected to mimic each brand of whisky analysed. When mixed with the Au^{3+} solution, whilst there was some reduction of the gold, there was little evidence of Au NPs forming to the same extent as the control whisky samples (Figure S12B and S12C) suggesting there are other shape control ligands we have not implemented here, and that reducing power is more than the sum of these parts.

Another congener chemical group identified by NMR was sugars such as glucopyranose and sucrose. Naturally occurring sugars such as fructose, glucose, lactose and mannose have previously been shown to reduce gold cations to Au NPs.[7] However this reaction requires basic conditions, and the pH of whisky is acidic at approximately pH 4 (brand dependent), meaning any naturally occurring sugars from the distillation and maturation process are not able to contribute to the overall reducing effect of whisky. This was confirmed when no reduction of Au^{3+} solution was observed when mixed with 40% EtOH (pH 4) spiked with either glucose or sucrose at concentrations (< 4 mM total) found from the NMR studies of the whisky (data not shown).

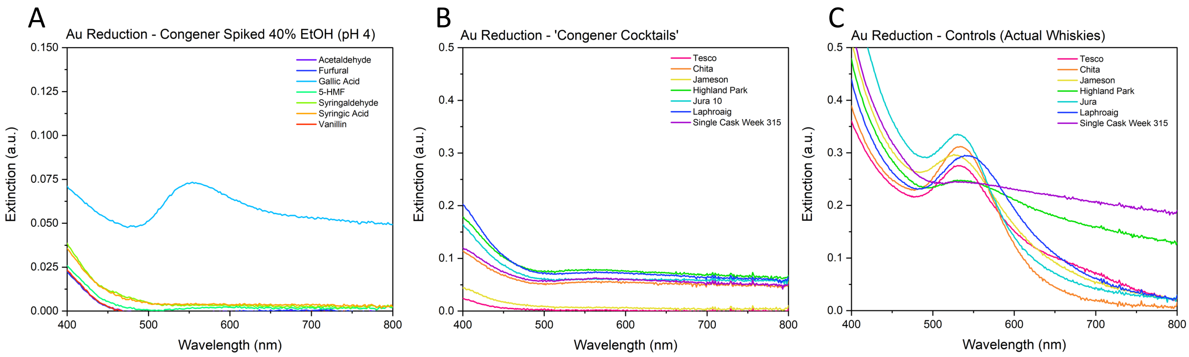

Figure S12 – Extinction spectra from mixing (A) Single congeners spiked into 40% EtOH pH 4, (B) Congener cocktails in 40% EtOH pH4 or (C) Control samples of the actual whiskies, with Au^{3+} solution as per the model method. Water background spectrum subtracted prior to plotting.

As gallic acid was the only solo congener to show some reducing ability after the 1-hour timepoint we hypothesised one of the unknown congeners that has not shown up in NMR analysis could be tannic acid.

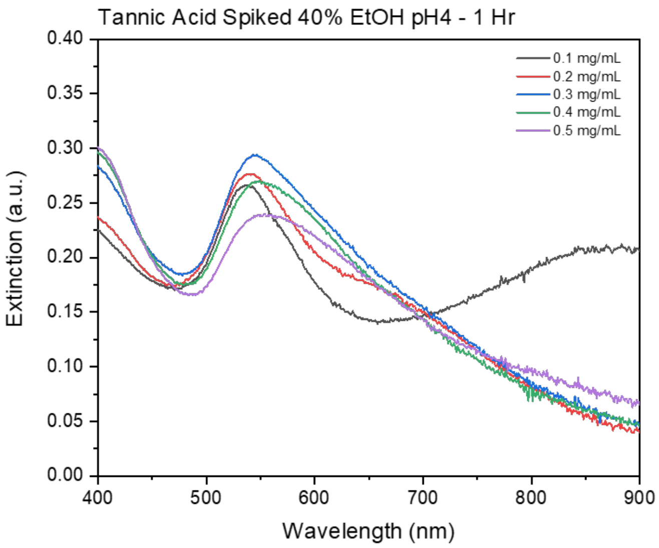

Figure S13 – Extinction spectra of Au NPs reduced by tannic acid spiked solutions of 40% EtOH (pH 4). Water background spectrum subtracted prior to plotting.

Tannins are a group of polyphenols commonly found in cask aged drinks such as wines and spirits, where those extracted from the oak casks are hydrolysable, with the monomer of many tannins being gallic acid, a congener identified in multiple whisky samples in the NMR studies. Although not easily detected by NMR (possibly latent in the broad peaks at ~6.5 – 7 ppm), tannic acid (a polyphenol), has previously been identified in whisky. Using literature values of 0.1 – 0.5 mg/mL,[3] tannic acid was spiked into a 40% ethanol solution (pH 4) and mixed with the Au^{3+} solution, leading to Au NP formation with a clear plasmon band (Figure S13). This suggests tannins and other low concentration polyphenol compounds, could be contributing congeners to the overall reducing potential of whisky. This may have useful implications for the sensor to be applied in peated and non-peated whiskies, where phenol concentrations vary dramatically.

Estimating the cost of the test

To approximate the cost of running the assay, it was calculated that for 100 µL (0.125 mM) of $HAuCl_4$ required approximately 4.25 µg of material. The $HAuCl_4$ was purchased at £133/g so this mass equates to a cost of £0.0006. The price of a cask of whisky varies enormously based on age, source, and cask size, but at a conservative estimate of £10,000 for an aged 200 L cask of whisky, then 50 µL of that whisky has a cost of £0.003, making the analyte the most expensive component in the assay.

References

(1) Quesenberry, M. S.; Lee, Y. C. A Rapid Formaldehyde Assay Using Purpald Reagent : Application under Periodation Conditions. *Anal. Biochem.* **1996**, *234* (1), 50–55.
(2) Chetrariu, A.; Dabija, A. Spent Grain from Malt Whisky : Assessment of The Phenolic Compounds. *Molecules* **2021**, *26* (11), 3236.
(3) Liebmann, A. J.; Scherl, B. Changes in Whisky While Maturing. *Ind. Eng. Chem.* **1949**, *41* (3), 534–543.
(4) Kew, W.; Bell, N. G. A.; Goodall, I.; Uhrín, D. Advanced Solvent Signal Suppression for the Acquisition of 1D and 2D NMR Spectra of Scotch Whisky. *Magn. Reson. Chem.* **2017**, *55* (9), 785–796.
(5) Kew, W.; Goodall, I.; Uhrín, D. Analysis of Scotch Whisky by 1H NMR and Chemometrics Yields Insight into Its Complex Chemistry. *Food Chem.* **2019**, *298*, 125052.
(6) Stockwell, M.; Goodall, I.; Uhrín, D. Quantification of Whisky Congeners by 1 H NMR Spectroscopy. *Anal. Sci. Adv.* **2020**, *1* (2), 132–140.
(7) Brasiunas, B.; Popov, A.; Ramanavicius, A.; Ramanaviciene, A. Gold Nanoparticle Based Colorimetric Sensing Strategy for the Determination of Reducing Sugars. *Food Chem.* **2021**, *351*, 129238.
